# Occlusio Pupillae: A Duet of Darkness Where the Patient Sees Naught, and the Doctor Discerns Not

**DOI:** 10.7759/cureus.63370

**Published:** 2024-06-28

**Authors:** Puja Hingorani-Bang, Meghana Kandi, Nidhi Mamtani, Vandana A Iyer

**Affiliations:** 1 Department of Ophthalmology, All India Institute of Medical Sciences, Nagpur, Nagpur, IND

**Keywords:** phacoemulsification, neodymium:yttrium-aluminum-garnet (nd:yag) laser applications, yag pupillary membranotomy, one-eyed patient, pseudo-bleb, glaucoma, uveitis, complicated cataract, phthisical eye, occlusio pupillae

## Abstract

A 71-year-old, one-eyed female patient presented with a loss of vision in the right eye due to trauma 20 years ago and a progressive diminution of vision in the left eye over the past six years. An ambiguous history of some surgery performed on the left eye was elicited, with no available records, adding an element of uncertainty to this case. Visual acuity (VA) was noted as no light perception (No PL) in the right eye and light perception with accurate projection of rays (PL+, PR accurate) in the left eye. Anterior segment slit-lamp evaluation of the right eye showed a shrunken globe with low intraocular pressure (IOP). The left eye exhibited signs of chronic uveitis with occlusio pupillae, non-visualization of the lens, and a doubtful conjunctival bleb with scleral thinning superior to the limbus. B-scan evaluation was suggestive of phthisis in the right eye and an equivocal lens shadow in the left eye.

A yttrium aluminum garnet (YAG) pupillary membranotomy was planned for the left eye under steroid cover and was cautiously attempted, successfully detaching the occlusio membrane and revealing an underlying complicated cataract beneath it. Post-laser, medical management included topical anti-glaucoma and steroid medications, along with systemic steroids. The VA improved from PL+, PR accurate to 3/60 (improving to 6/60 with a Retinal Acuity Meter). After stabilization of the uveitis over the next few weeks and under a steroid cover, a temporal clear-corneal phacoemulsification was cautiously performed with intra-operative management of the small pupil, and a hydrophobic lens was implanted. At one month post-surgery, the patient’s best-corrected visual acuity had improved to 6/12 for distance and N6 for near.

This report highlights a compelling instance wherein the neodymium:Yttrium-aluminum-garnet (Nd:YAG) laser was efficaciously employed for a lesser-known application in resolving a diagnostic dilemma and for instituting an interim treatment strategy in a challenging case involving a one-eyed patient prior to planning a definitive surgery.

This case emphasizes the importance of thinking out of the box, ensuring comprehensive preoperative and careful intra-operative precautions in the management of patients diagnosed with complex ocular inflammatory conditions, so as to optimize visual outcomes, eventually resulting in achieving a gratifying reduction of visual disability and improvement of quality of life.

## Introduction

Chronic anterior uveitis is recognized for its potential to cause a spectrum of anterior and posterior segment complications, notably posterior synechiae, occlusio pupillae, complicated cataract, secondary glaucoma, and cystoid macular edema [1].

The advent of the neodymium:Yttrium-aluminum-garnet (Nd:YAG) laser technology in 1964 has ushered in diverse applications within Ophthalmology. Notable indications include capsulotomy of the posterior capsule to address opacification subsequent to cataract surgery and peripheral iridotomy in angle-closure glaucoma.

This report highlights a compelling instance wherein Nd:YAG laser was efficaciously employed for the treatment of the inflammatory pupillary membrane in a 71-year-old unilaterally sighted female presenting with occlusio pupillae and posterior synechiae secondary to chronic uveitis [2].

The utilisation of Nd:YAG laser in this context demonstrates its evolving and unconventional role in ophthalmic interventions, providing a minimally invasive approach to address intricate ocular sequelae associated with chronic inflammatory conditions. The successful outcome in this case underscores the potential benefits of Nd:YAG laser in mitigating vision-threatening complications, emphasizing the importance of tailored interventions in the management of complex ocular pathologies [3].

## Case presentation

History

A 71-year-old, one-eyed female patient presented to our outpatient department in September 2023 with the chief complaints of loss of vision in her right eye 20 years ago and progressive diminution of vision in her left eye for six years.

She had a past history of trauma to the right eye with vegetative matter over 20 years ago, followed by redness, pus discharge, whitish opacity, and eventual loss of vision. She also gave a vague past history of some surgery performed on the left eye, details or records of which were not available.

There was no history of trauma, fever, joint pains, oral ulcers, chronic cough, dyspnea, or other systemic illnesses.

Examination

On examination, her vitals were stable. The rest of the systemic evaluation was within normal limits.

Ophthalmic Evaluation

The patient's visual acuity was reduced to no perception of light (No PL) in the right eye and perception of light with accurate projection of rays in the left eye (PL+, PR accurate). The IOP was low-unrecordable in the right eye and 10 mmHg in the left eye.

**Figure 1:**
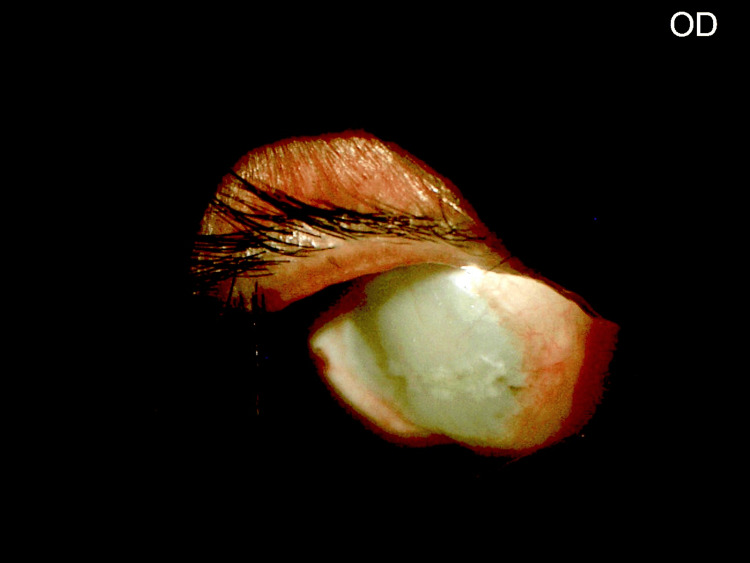
Phthisical right eye (slit lamp examination).

**Figure 2:**
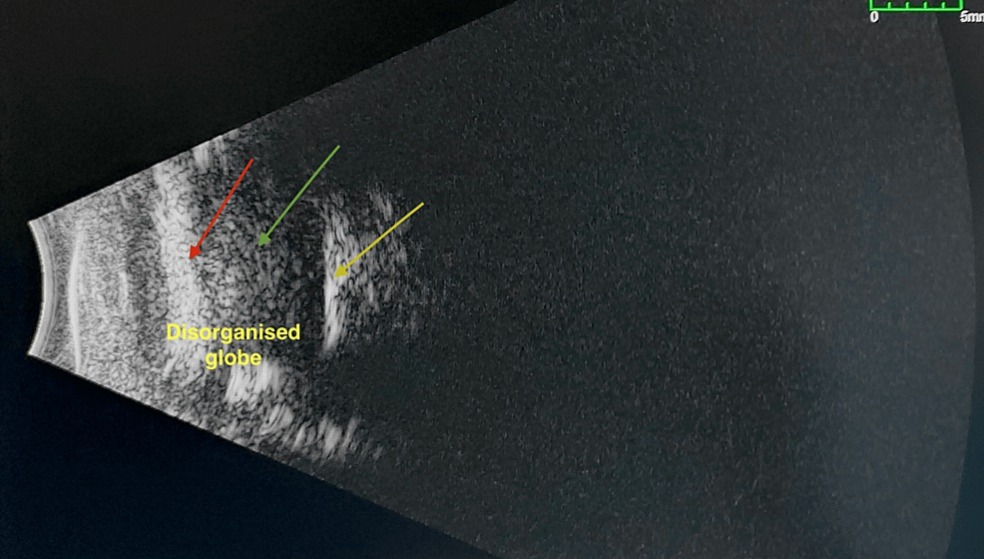
B-scan RE (phthisical shrunken eye). Disorganized globe is seen. The vitreous (green arrow) is full of dot echoes due to a possible old vitreous hemorrhage. Intrascleral calcification is seen (yellow arrow). The lens complex echo is not clearly discernible (red arrow). RE: Right Eye; B-scan: Brightness Scan.

Based on slit lamp and B-scan evaluation, a diagnosis of phthisis bulbi in the right eye was made (Figures 1-2).

*Left Eye Findings* 

**Figure 3:**
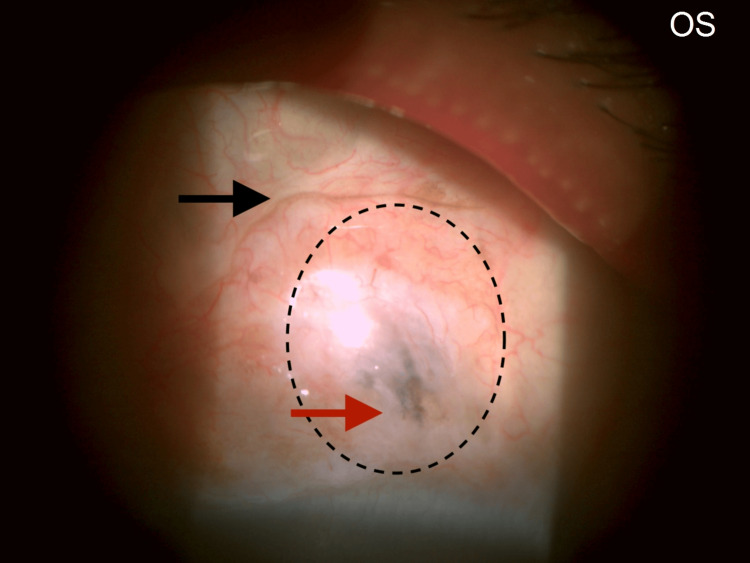
Bleb-like elevation superior to the limbus. The dotted outline corresponds to the bleb-like elevation superior to the limbus. The black arrow points to the pigmentary line demarcating the bleb margin. The red arrow points to the pigmented uveal tissue seen through an area of localized scleral thinning. Differential Diagnoses: (1) Secondary to a possible prior Mitomycin-C application, or (2) An early or evolving intercalary staphyloma. (However, there was a doubtful history of surgery and no definitive history of trauma in the past).

**Figure 4:**
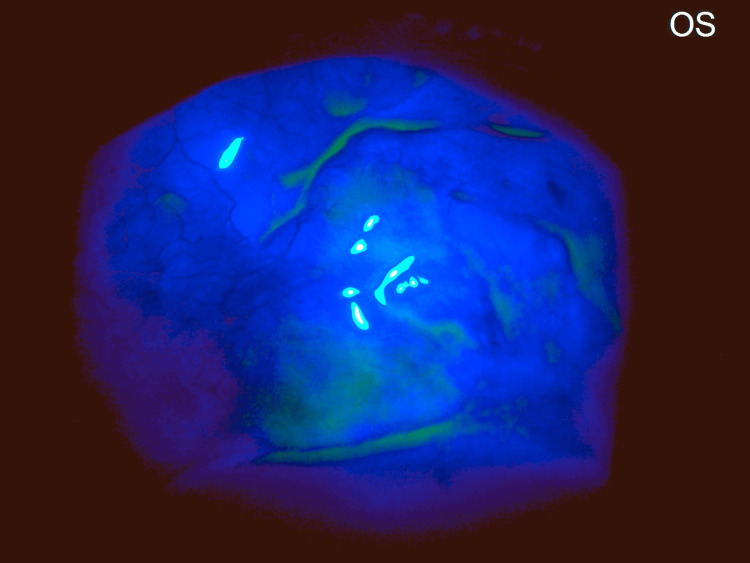
Seidel's test negative. Some pooling of fluorescein dye is observed along the superior and inferior margins of the bleb.

Conjunctiva: A bleb-like elevation (?pseudo-bleb) was noted superior to the limbus. It was translucent, with a pigmentary line of deposit along its superior border, and underlying pigmentary uveal tissue was seen through a localized area of scleral thinning (?Mitomycin-C induced or ?due to an evolving/early intercalary staphyloma) (Figure 3). Seidel's test was negative (Figure 4). With a vague, equivocal history of past surgery and the absence of a definitive history of trauma, however, the etio-pathogenesis remained uncertain.

Cornea: Clear.

**Figure 5:**
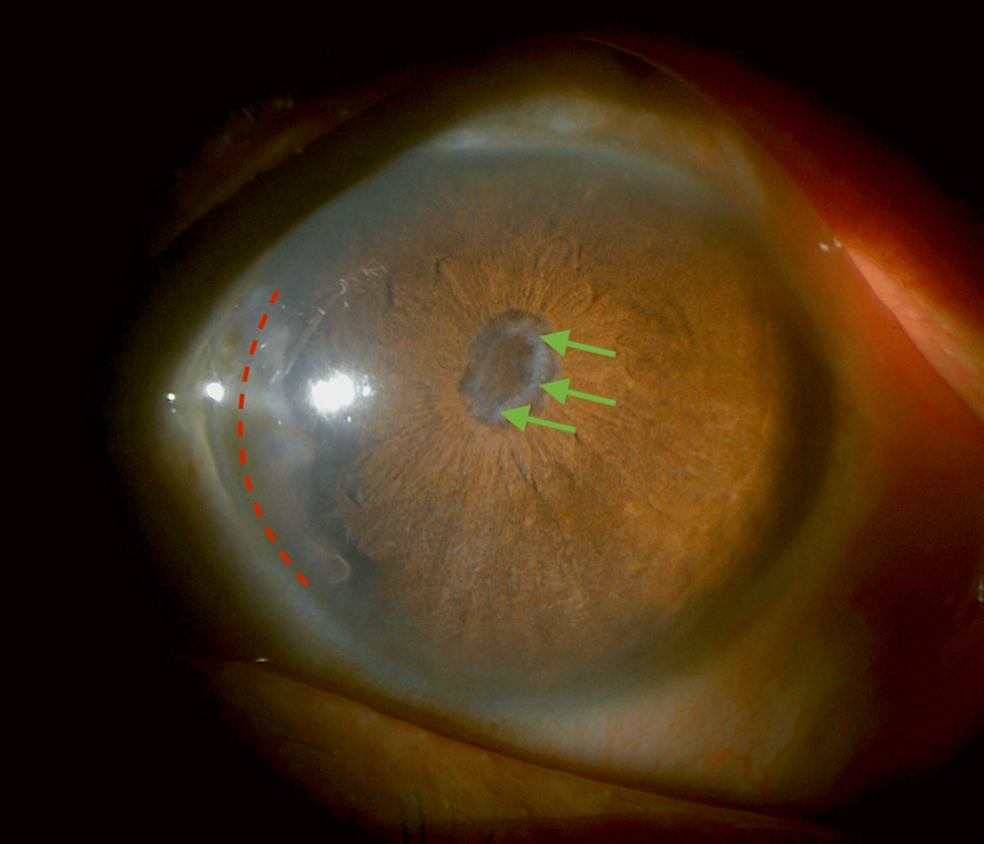
Anterior segment left eye. Broad anterior synechiae with irido-corneal adhesion from 7 to 10 o'clock nasally (broken red line demarcating the extent of adhesion). Occlusio pupillae (green arrows).

**Figure 6:**
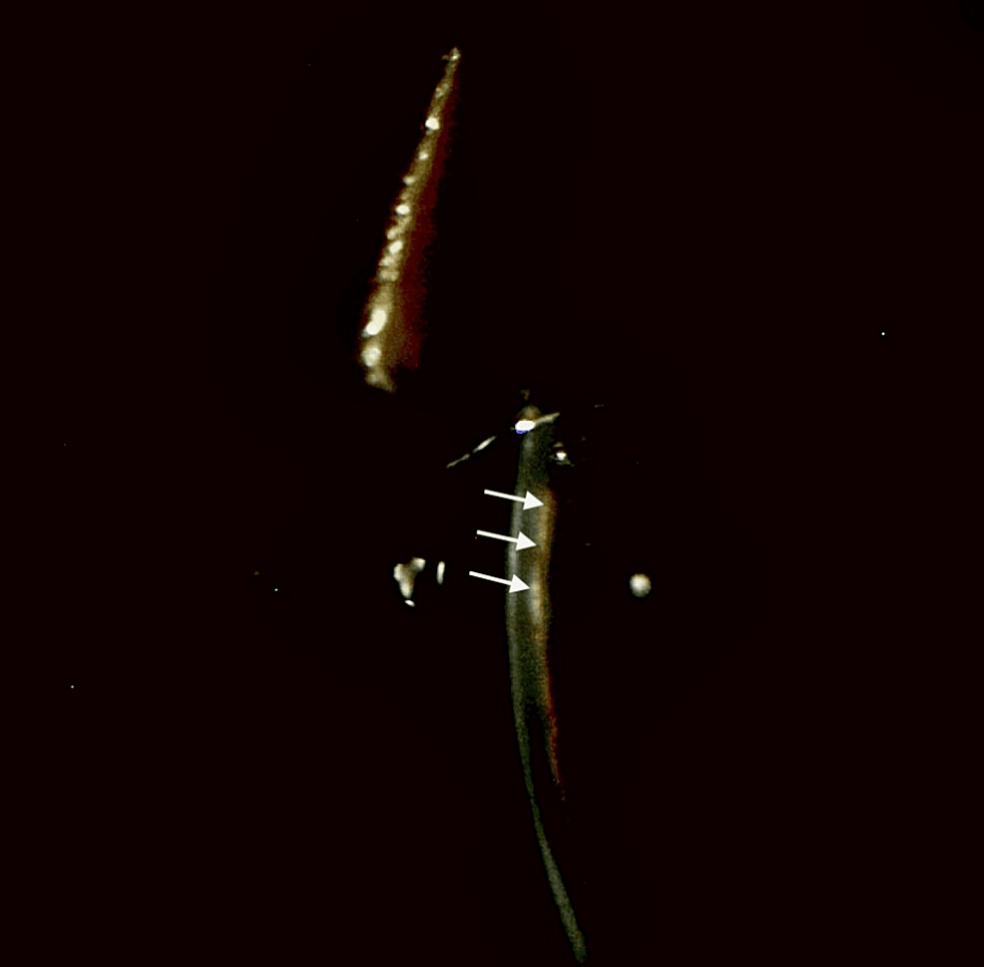
LE: Nasal slit beam showing irido-corneal adhesion. Iridocorneal adhesion is seen as an irregularity on the posterior corneal surface (white arrows). LE: Left eye.

Anterior chamber: Depth was Van Herick grade 2 with peripheral anterior synechiae seen nasally from 7 to 10 o'clock, suspected to be due to an old scarred side port or due to the uveitic process (Figures 5-6).

**Figure 7:**
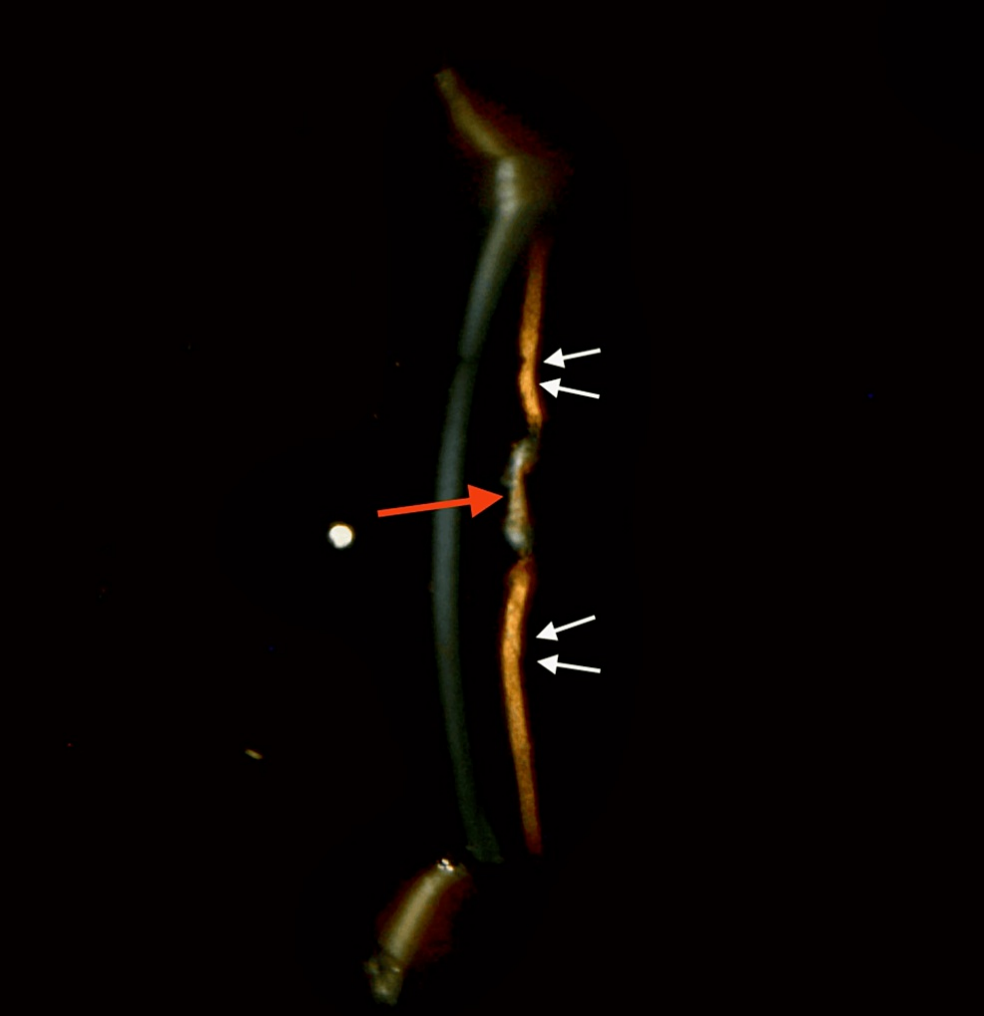
LE: Slit beam showing occlusio pupillae. Pupillary membrane (red arrow) with early iris bombe (white small arrows). LE: Left eye.

Iris: Washed-out appearance with occlusio pupillae and a non-dilating pupil was seen, making it difficult to determine the status of the lens and posterior segment (Figures 5, 7).

Pupil: A non-dilating pupil with a total pupillary membrane was seen obstructing the view of the lens, with an inability to discern clinically whether an intraocular lens (IOL) or a crystalline lens was behind it (especially because the patient gave a history of some form of surgery performed in the left eye 10 years ago).

**Figure 8:**
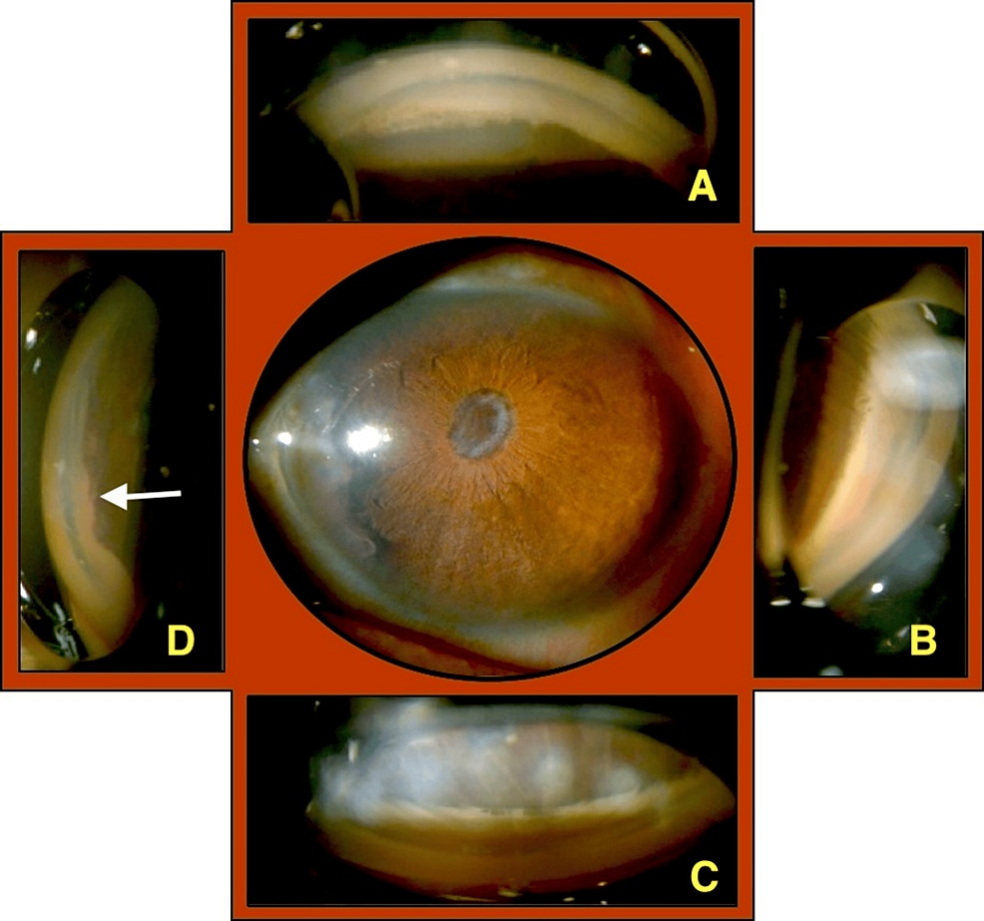
LE: Gonioscopy findings. No trabeculectomy stoma seen superiorly (panel A). Anterior synechiae and adhesions seen nasally (panel D: white arrow). LE: Left eye.

Gonioscopy: Showed irido-trabecular contact and high peripheral anterior synechiae nasally, showing no evidence of a trabeculectomy stoma superiorly adjacent to the bleb-like formation (Figure 8).

**Figure 9:**
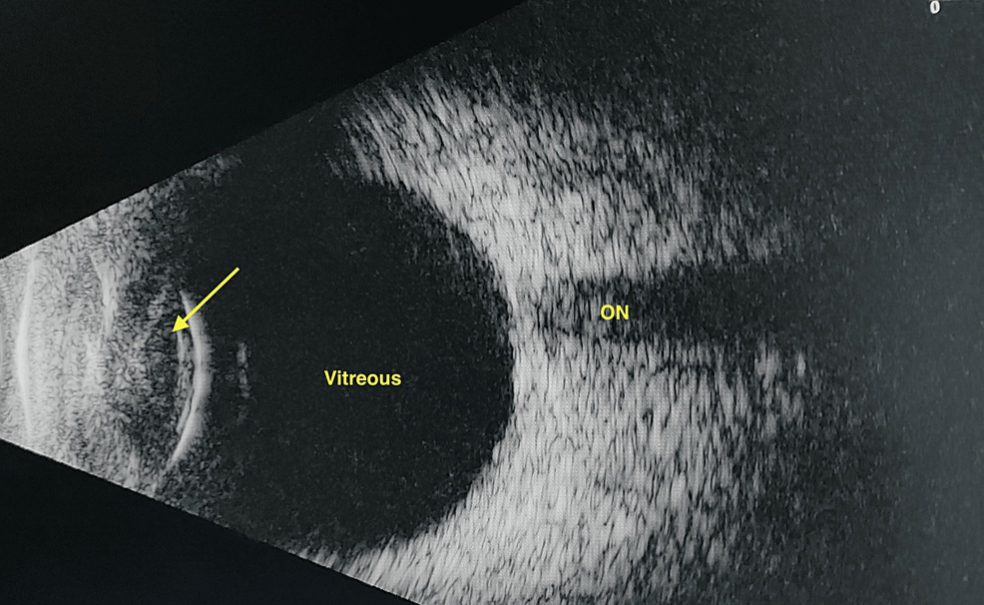
B-scan: LE. The optic nerve (ON) shadow is normal. The vitreous shows a few reflective echoes. The globe is organized. The capsular bag is seen, but a definitive IOL versus lens echo is not discerned (yellow arrow). LE: Left eye; IOL: Intraocular lens.

B-scan: Definitive lens echo/IOL reverberations were not noted; the vitreous showed a few low-reflective dot echoes, and the retina was attached with a normal optic nerve head shadow (Figure 9).

**Figure 10:**
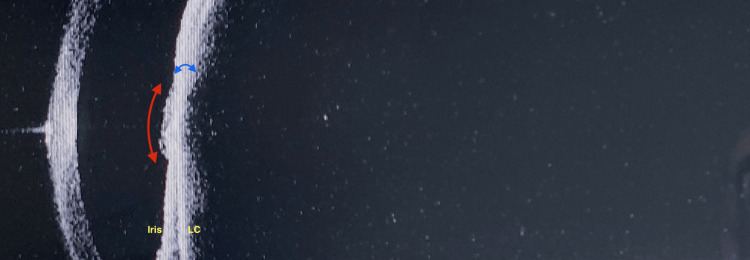
LE: AS SS-OCT: on presentation. A slightly bulging pupillary membrane (red curved double arrow) is seen adherent to the pupil centrally. The iris and anterior lens capsule (LC) cannot be optically differentiated anteriorly in the region around the membrane (blue double arrow). Posterior structures are not visible. LE: Left eye; AS: Anterior segment; SS-OCT: Swept-source optical coherence tomography.

Anterior segment optical coherence tomography (AS-OCT): Initial scan findings showed a blocked pupil, a bulging pupillary membrane, wide iridolenticular contact with non-visualization of the posterior segment structures (Figure 10).

Management

Routine investigations, namely, hemoglobin 15.2, total leukocyte count (TLC) 9.07, platelets 390,000, erythrocyte sedimentation rate (ESR) 37, liver function test/kidney function test (LFT/KFT) were normal; rheumatoid arthritis (RA) factor was negative, antinuclear antibody (ANA)-negative, and viral markers were non-reactive as part of our systemic uveitis work-up.

The patient was referred to a physician to rule out systemic associations of uveitis and for oral steroid clearance. No specific systemic cause could be ascertained. She received systemic clearance to start oral steroid prophylaxis to plan for any upcoming invasive intervention, in view of the uveitis. Cystoid macular edema (CME) is one of the potential complications associated with uveitis, and surgical procedures may exacerbate inflammation, increasing the risk of CME and uveitis recurrence.

Procedure

**Figure 11:**
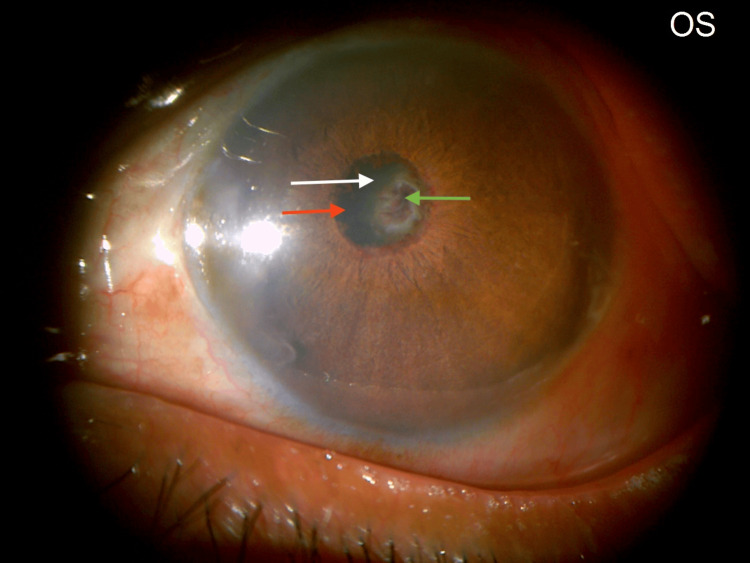
LE: post-YAG membranotomy. The pupillary membrane has been released from the pupil along 360 degrees of its margin. An iris shadow (red arrow) is seen on the cataractous lens (white arrow). A central shriveled tag of the pupillary membrane is visible on the anterior lens capsule (green arrow). LE: Left eye; YAG: Yttrium Aluminum Garnet.

**Figure 12:**
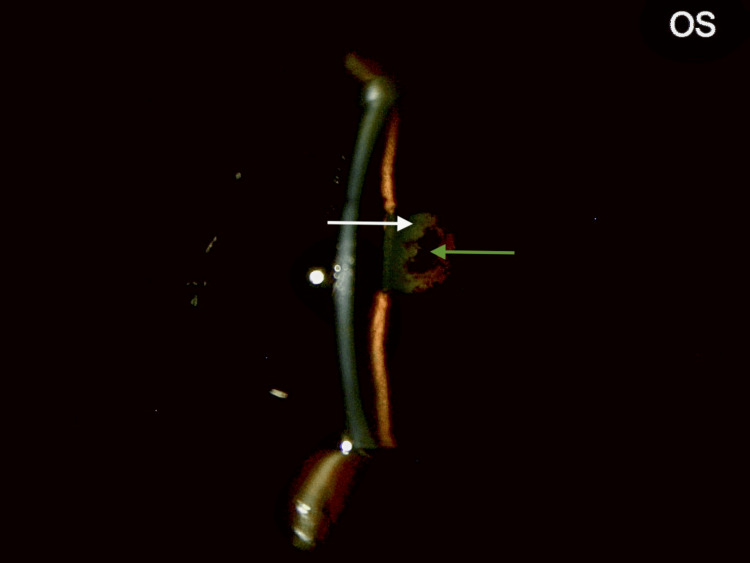
LE: slit beam view post YAG membranotomy. A cataractous lens (white arrow) is seen with remnants of the pupillary membrane (green arrow). LE: Left eye; YAG: Yttrium Aluminum Garnet.

A trial of left eye YAG membranotomy under topical anesthesia was planned. Left eye YAG laser membranotomy was performed under topical anesthesia. An anterior de-focus was applied. Low energy settings were used. These were titrated upwards until membrane disruption was seen. Shots were fired along 360 degrees, achieving the desired result without causing any lens damage or bleeding. The parameters used were: an anterior offset on the YAG machine; a total of 60 shots ranging from 0.7 to 1.8 mJ per shot, and a total utilized energy of 90.2 mJ. Post-procedure, slit lamp evaluation of the left eye revealed a complete detachment of the occlusion membrane from the entire circumference of the pupillary margin. The membrane was seen to be attached in the center to what was surprisingly revealed to be not an IOL but the anterior lens capsule of a complicated cataract (Figures 11-12).

**Figure 13:**
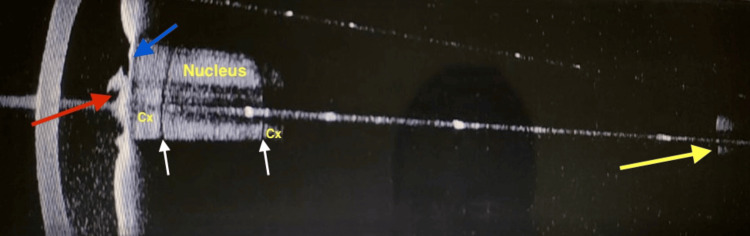
LE: anterior segment SS-OCT after YAG membranotomy. A tag of the membrane is seen attached (red arrow) to the thickened anterior lens capsule (blue arrow). The pupil appears larger, and lens details are more clearly seen than earlier (Figure 10). The dense nucleus is separated from the anterior and posterior cortex (Cx) by a clear, lucent zone (white arrows). The beam travels to the retina (yellow arrow). LE: Left eye; SS-OCT: Swept-Source Optical Coherence Tomography; YAG: Yttrium Aluminum Garnet.

AS-OCT (Figure 13) and B-scan repeated post-procedure confirmed the presence of a crystalline cataractous lens in the left eye. A regimen of systemic and topical steroids, aqueous suppressants, and cycloplegics was prescribed in the left eye for 2 weeks [4]. Cataract surgery was planned. Pre-operative conjunctival swabs were sent; a cautionary protocol we follow for one-eyed patients, which were found to be culture negative.

Outcome of YAG Procedure

At one week post-procedure, the visual acuity in the left eye improved to counting fingers at 3 meters, up from a baseline of PL+, PR accurate. With potential acuity meter (PAM) testing, it improved further to 6/60. No spikes were noted in the IOP.

Surgical intervention

A temporal phacoemulsification was performed under steroid cover, after ensuring that the eye was quiet for at least 2 months prior to surgery. Intraoperative challenges included: (1) the superior pre-existing bleb with scleral thinning, justifying the temporal, clear-corneal incision, and (2) the non-dilating pupil, tackled with iris hooks and high molecular weight ocular viscoelastic devices. Following the release of posterior synechiae, an adequately sized rhexis of 5-6 mm was made, and controlled phacoemulsification was performed. An in-the-bag, hydrophobic lens was successfully placed.

Postoperative Course

**Figure 14:**
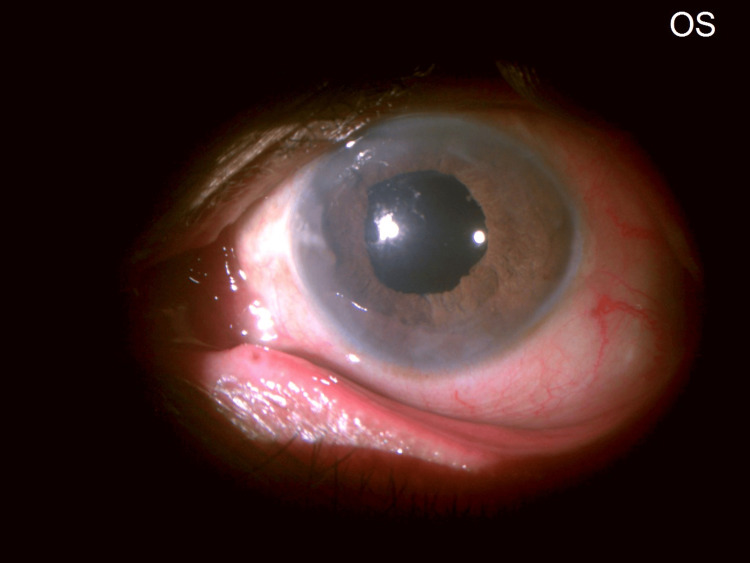
LE: post-cataract surgery: pseudophakia, slightly irregular pupil. LE: Left eye.

**Figure 15:**
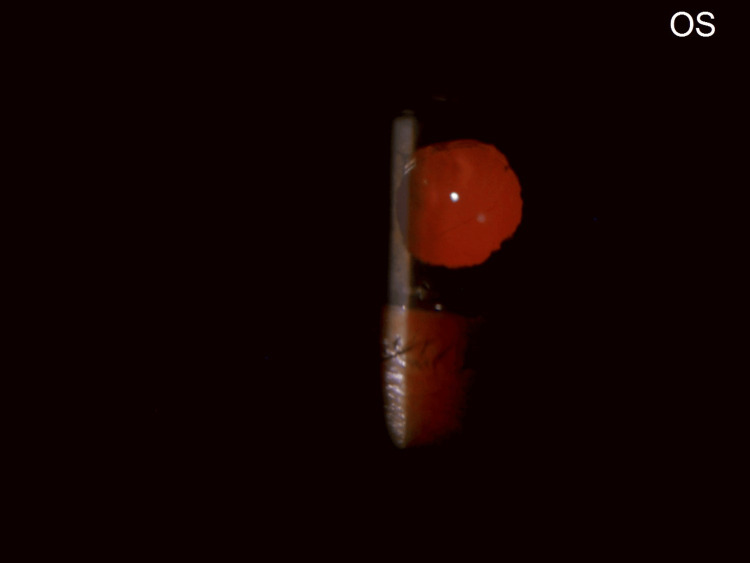
LE: post cataract surgery: pseudophakia with red glow. LE: Left eye.

On post-operative day 1, the left eye wound was healthy. There was a clear cornea, well-formed anterior chamber with Grade 1-2 cells, central irregular pupil, centrally placed posterior chamber IOL, and a red pupillary glow (Figures 14-15).

**Figure 16:**
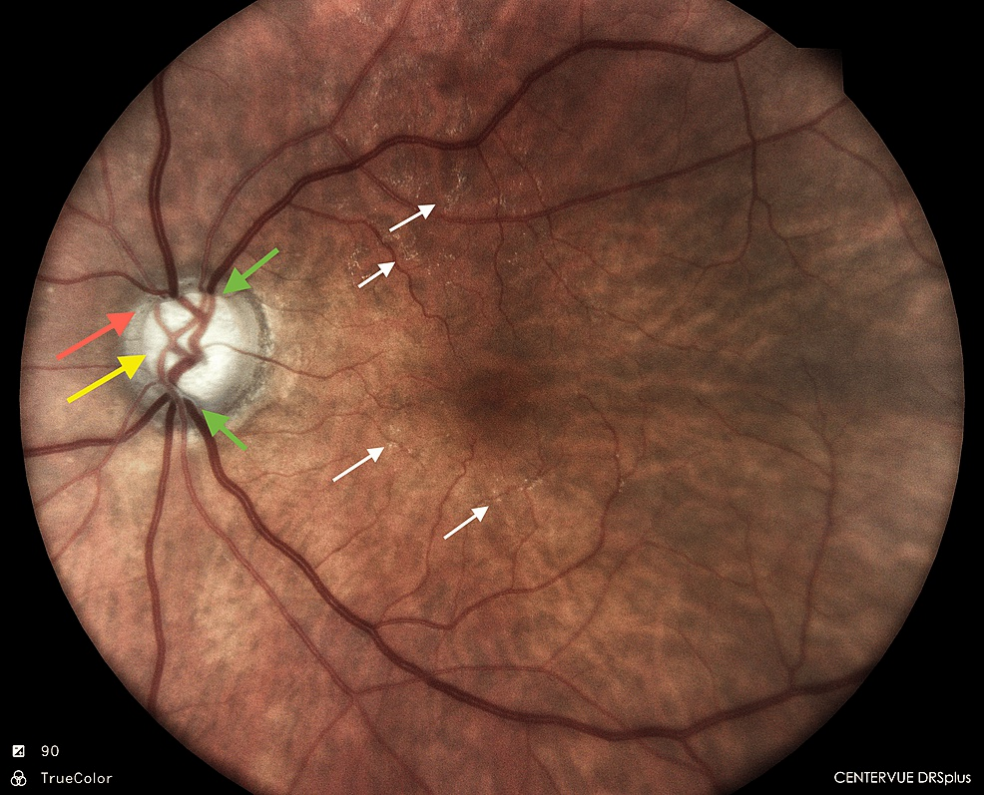
LE: Fundus (post-cataract surgery). A CD ratio of 0.8 is observed (yellow arrow). The cup appears deep. The neuro-retinal rim is pale (red arrow) with thinning of the superior and inferior rims (green arrows). Some retinal exudates are visible near the arcades (small white arrows). LE: Left eye; CD ratio: Cup-to-disc ratio.

The first view of the fundus since presentation revealed a cup-to-disc ratio (CDR) of 0.8:1 with superior and inferior rim thinning (Figure 16). A dull foveolar reflex was seen with tessellation in the periphery. The patient was kept on topical steroids and antibiotic eye drops along with one anti-glaucoma medication on the advice of our glaucoma colleague (Brimonidine 0.1% BD).

The patient’s best corrected visual acuity (BCVA) in the left eye, with resolving iritis on post-operative day 3, was 6/24. The patient was able to perform her daily routine activities independently and expressed psychological relief from her previously totally dependent and blind life.

At post-operative one month, her eye was quiet, and the BCVA was 6/12 with +0.50 D Sph/-1.00 cyl x 80° for distance and N6 with an add of +2.50 D Sph for near.

## Discussion

One of the notable complications of long-standing uveitis is the development of pigmented membranes across the pupil [5] and secondary glaucoma, both requiring vigilant lifelong surveillance and treatment. Yet, the most commonly performed definitive surgical intervention in uveitis cases is often cataract surgery for visual rehabilitation, as in our case. Cataract surgery, typically performed under steroid cover and with appropriate precautions, aims to restore transparency of the ocular media, thereby improving vision in these cases.

This specific case is noteworthy due to its distinctive aspects. Firstly, this was a one-eyed patient with extremely poor vision in her only functioning eye, presenting with occlusio pupillae that obscured the clinician’s ability to assess the status of the lens and posterior segment. Further posing a significant diagnostic and therapeutic challenge was an inconclusive B-scan, a suspicious bleb with thinned-out sclera, pitted against a puzzling past surgical history: possibly of cataract and/or glaucoma surgery, but with no records whatsoever.

Despite these impediments, a successful attempt at pupillary membranectomy, with the help of the minimally-invasive, low-setting Nd-YAG laser at 0.7-1.8 mJ per shot using sixty pulses was made. This resulted in only a mild anterior chamber reaction, no damage to the underlying lens or IOP spikes, helping to relieve the pupillary block, improve vision, and to determine the lens and posterior segment status.

Some previous reports have documented the efficacy of utilizing single pulses of YAG laser ranging from 4 to 12 mJ to address post-surgical or uveitic inflammatory membranes, iridolenticular (posterior) synechiae, and small pupils. This laser is advantageous in that it does not require pigmentation of the target. It is a Q-switched, infra-red, solid-state laser of wavelength 1024nm. A forceful rupture of adhesions can be achieved as it is a photo-disruptive laser. For any laser, emitted photons are in phase, producing monochromatic, polarized, coherent high-intensity light [3,6].

Steinert reported the use of the Nd:YAG laser at 4 mJ per pulse to perform a pupillary membranectomy in a pseudophakic eye with anterior chamber intraocular lens (ACIOL), having a pupillary block following endophthalmitis [6].

Gandham SB et al. described a series of seven pseudophakic eyes with post-operative inflammatory pupillary membranes treated with Nd:YAG laser at 1.2 to 3.1 mJ per shot, using an average of 48.3 ± 20.1 pulses per procedure [7].

Pupilloplasty or coreoplasty involves cutting through the iris stroma or the pupillary sphincter to open up an obstructed visual axis. Steinert reported a case of pseudophakia with seclusio pupillae, where an energy of 6 mJ per pulse was used to successfully perform a coreoplasty through an Abraham lens [6]. Sastry P similarly described Nd:YAG pupilloplasty in four cases of occlusio pupillae using 30-45 shots of 3 mJ per shot in a staged manner over 2-3 weeks; two of these eyes were pseudophakic [8].

Kim EA et al. described a series of 114 eyes that had undergone some form of intra-ocular surgery, predominantly vitrectomy, who presented with synechiae. Nd:YAG synechiolysis was performed in 21 eyes with synechiae limited to 1 clock hour and to the pupillary sphincter alone. Shots of 0.6 to 1.5 mJ energy were used following the instillation of a mydriatic-cycloplegic to expose the synechiae; three eyes had recurrence needing re-laser [9].

Khurana G et al. described a case of bilateral occlusio pupillae with ring synechiae where Nd:YAG synechiolysis was attempted with energy settings of 0.6 to 1.5 mJ per shot [3]. They were able to achieve success in one eye and only partial success in the other, despite multiple repeated attempts spanning five weeks.

Among other less-commonly employed indications, Griener E and Lambert SR used an energy of 2 mJ to successfully treat tractional corectopia in a child with an anterior membrane strand [10], whereas Hurvitz LM used the Nd:YAG laser in a series of pseudophakic eyes with anterior capsular phimosis following small capsulorrhexis openings [11].

Complications of Nd:YAG laser for these relatively infrequent and anterior indications may include elevated intraocular pressure, IOL damage, complicated cataract formation [3,8], iritis, hyphema, and corneal burns. Low power settings, with due caution and proper offsets, must be exercised in phakic patients who are at risk of developing lenticular opacity following such procedures. Medical interventions, including anti-inflammatory medications and glaucoma management, are crucial adjuncts to stabilize the inflammation post-procedure.

## Conclusions

A holistic approach encompassing comprehensive patient history, clinical assessments, modern diagnostic tools, adequate referrals, medical interventions, YAG laser procedures, and, if necessary, surgical interventions can contribute to restoring vision for individuals labeled as visually impaired or even blind, enabling them the gratification of resuming their daily activities independently.

In this case, out-of-the-box thinking, innovative use of the laser, a team approach by the treating anterior-segment ophthalmologist with the rheumatologist (internist) and the glaucoma colleague, and finally, a carefully planned cataract surgery with IOL implantation, helped in managing the condition with good visual outcomes and significant patient satisfaction.
